# Crystal structure of *tert*-butyl 4-[4-(4-fluoro­phen­yl)-2-methyl­but-3-yn-2-yl]piperazine-1-carboxyl­ate

**DOI:** 10.1107/S2056989021002346

**Published:** 2021-03-05

**Authors:** Ashwini Gumireddy, Kevin DeBoyace, Alexander Rupprecht, Mohit Gupta, Saloni Patel, Patrick T. Flaherty, Peter L. D. Wildfong

**Affiliations:** a Graduate School of Pharmaceutical Sciences, Duquesne University, 600 Forbes Ave, Pittsburgh, PA 15282, USA; b Department of Chemistry, Duquesne University, 600 Forbes Ave, Pittsburgh, PA 15282, USA

**Keywords:** crystal structure, quaternary carbons, propargyl­amine

## Abstract

The title sterically congested piperazine derivative, C_20_H_27_FN_2_O_2_, was prepared using a modified Bruylants approach. A search of the Cambridge Structural Database identified 51 compounds possessing an *N-tert-*butyl piperazine substructure. Of these only 14 were asymmetrically substituted on the piperazine ring and none with a synthetically useful second nitro­gen. Given the novel chemistry generating a pharmacologically useful core, determination of the crystal structure for this compound was necessary. The piperazine ring is present in a chair conformation with di-equatorial substitution. Of the two N atoms, one is *sp* ^3^ hybridized while the other is *sp* ^2^ hybridized. Inter­molecular inter­actions resulting from the crystal packing patterns were investigated using Hirshfeld surface analysis and fingerprint analysis. Directional weak hydrogen-bond-like inter­actions (C—H⋯O) and C—H⋯π inter­actions with the dispersion inter­actions as the major source of attraction are present in the crystal packing.

## Abstract 2

A sterically congested piperazine derivative, *tert*-butyl 4-[4-(4-fluoro­phen­yl)-2-methyl­but-3-yn-2-yl]piperazine-1-carboxyl­ate, was prepared using a modified Bruylants approach. Its novel chemistry with a synthetically useful second nitro­gen atom on the *N-tert-*butyl piperazine substructure generates a pharmacologically useful core.

## Chemical context   

**Figure 1:**
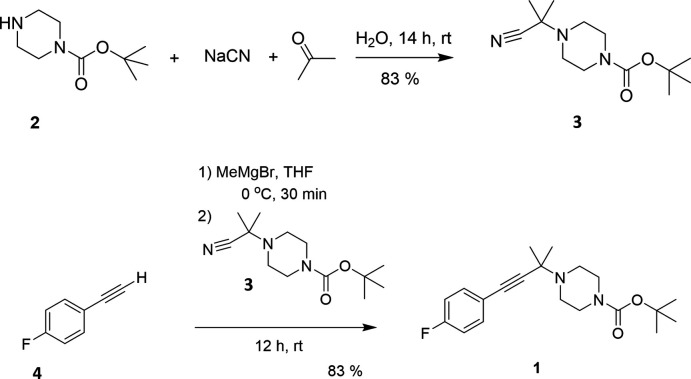
Synthesis of *tert*-butyl 4-[4-(4-fluoro­phen­yl)-2-methyl­but-3-yn-2-yl]piperazine-1-carboxyl­ate (**1**) *via* Bruylants reaction (Firth *et al.*, 2016 ▸).

In the course of designing novel sigma-2 ligands, it was necessary to synthesize 1-(2-methyl-4-phenyl­butan-2-yl)pip­erazines. These could be prepared in several steps from the corresponding alkyne **1** shown in Fig. 1 ▸. The challenge of synthesizing quaternary carbons (Wei *et al.*, 2020 ▸; Liu *et al.*, 2015 ▸; Volla *et al.*, 2014 ▸; Fuji, 1993 ▸; Martin, 1980 ▸), particularly amine-bearing quaternary carbons (Zhu *et al.*, 2019 ▸; Yeung *et al.*, 2019 ▸; Xu *et al.*, 2019 ▸; Velasco-Rubio *et al.*, 2019 ▸; Vasu *et al.*, 2019 ▸; Trost *et al.*, 2019 ▸; Ling & Rivas, 2016 ▸; Hager *et al.*, 2016 ▸; Clayden *et al.*, 2011 ▸; Fu *et al.*, 2008 ▸; Riant & Hannedouche, 2007 ▸), is well established. The presence of the *N*-*gem*-dimethyl group of **1** presented a significant synthetic challenge arising from steric congestion. Nucleophilic attack by an organometallic reagent into a transient 1-*N*-ethyl­idenepiperazinium has a literature precedent, but nucleophilic attack into the more sterically congested 1-*N*-propyl­idenepiperazinium inter­mediate by an alkynyl Grignard reagent is presented here for the first time. Four potential synthetic routes were identified including Katritzky benzotriazole trapping of an iminium (Monbaliu *et al.*, 2013 ▸; Ingram *et al.*, 2006 ▸; Katritzky, 1998 ▸; Katritzky *et al.*, 1989 ▸, 1991 ▸, 2005 ▸; Katritzky & Rogovoy, 2003 ▸; Katritzky & Saczewski, 1990 ▸), a Bruylants (Bruylants, 1924 ▸) trapping of an iminium, sequential addition of two methyl groups into an amide, and rearrangement to the *gem*-dimethyl group. All in-house attempts at the Katritzky benzotriazole (Tang *et al.*, 2013 ▸; Pierce *et al.*, 2012 ▸; Albaladejo *et al.*, 2012 ▸) or triazole (Prashad *et al.*, 2005 ▸) reactions failed. A variation on the Bruylants reaction (Liu *et al.*, 2014 ▸; Beaufort-Droal *et al.*, 2006 ▸; Prashad *et al.*, 2005 ▸; Kudzma *et al.*, 1988 ▸; Bernardi *et al.*, 2003 ▸) described herein was successful. The traditional Bruylants reaction captures a trapped iminium as the corres­ponding α-amino nitrile. In a subsequent reaction, the α-amino nitrile transiently forms an iminium that is then trapped with excess Grignard reagent. Conversion of the terminal alkyne **4** to the corresponding magnesiobromide acetyl­ide proceeded under established conditions. Attack of an alkynyl magnesium bromide into the transient iminium is precedented to yield tertiary carbon products. Generation of a quaternary carbon product in an analogous manner has not been described. A single paper details addition of a copper acetyl­ide into a Brulyants adduct (Tang *et al.*, 2013 ▸). Given the pharmacological importance of this compound and its tractable synthesis with novel chemistry, careful structural characterization by X-ray crystallographic analysis was necessary. Optimization of this reaction, subsequent structural elaboration, and specific pharmacological relevance will be detailed in later publications.
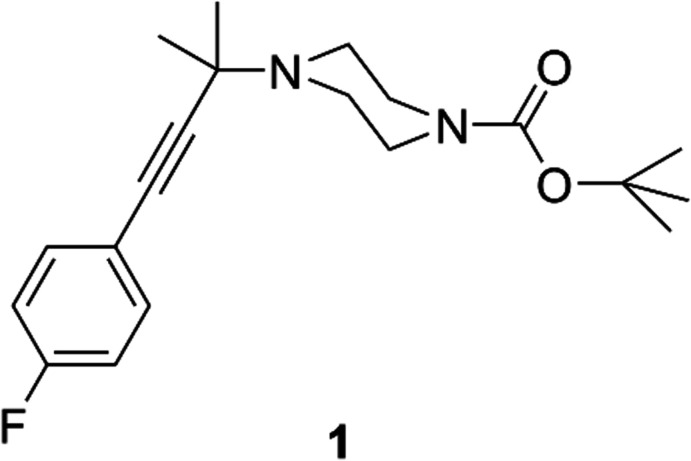


## Structural commentary   

**Figure 2:**
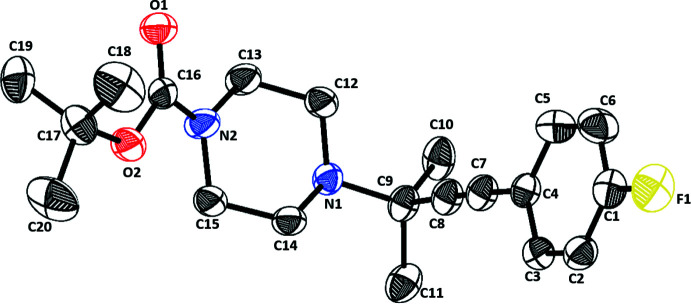
30% probability ellipsoid plot for the crystal structure solution of *tert*-butyl 4-[4-(4-fluoro­phen­yl)-2-methyl­but-3-yn-2-yl]piperazine-1-carboxyl­ate. Hydrogen atoms are omitted for clarity.

**Figure 3:**
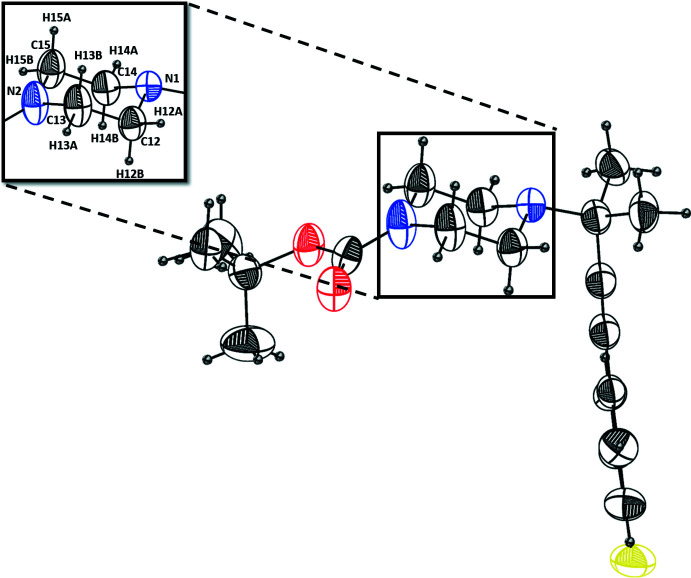
40% probability plot of the mol­ecular crystal structure solution of *tert*-butyl 4-[4-(4-fluoro­phen­yl)-2-methyl­but-3-yn-2-yl]piperazine-1-carboxyl­ate showing the l-shaped structure and the chair conformation of the piperazine ring.

The title compound, prepared from achiral reagents as a racemic mixture, crystallizes in the chiral monoclinic space group *P*2_1_ with one mol­ecule in the asymmetric unit as shown in the Scheme and Fig. 2 ▸. No heavy atoms are present in the structure and data were collected using Mo Kα radiation. Thus, the absolute structure of the randomly chosen crystals could not be determined reliably (Parsons *et al.*, 2013 ▸; Zhou *et al.*, 2015 ▸). In the mol­ecule, the NC(=O)O group of the carbamate exists in resonance. The bond lengths between carbon and other atoms (Table 1 ▸) are in the expected ranges with the bond length between O1—C16 [1.211 (3) Å] being the shortest, followed by N2—C16 [1.336 (3) Å], O2—C16 [1.345 (3) Å], and F1—C1 [1.359 (3) Å] owing to the presence of the more electronegative atoms oxygen, nitro­gen and fluorine. The bond length between C1—C6 [1.351 (4) Å] is the shortest among all the bond lengths in the phenyl group, possibly due to the inductive effect of fluorine. The spatial distance between the extreme atoms of propargyl­amine groups (C7⋯N1) was observed to be 3.508 (3) Å, which is slightly longer than for the other reported propargyl­amines (3.372–3.478 Å; Marvelli *et al.*, 2004 ▸; Sidorov *et al.*, 1999 ▸, 2000 ▸), and possibly due to the open L-shaped structure of the mol­ecule. Also, the piperazine ring is shown in its most stable chair form conformation in Fig. 3 ▸, as evidenced by the bond angles (Table 1 ▸) between N1—C12—C13 [110.77 (19)°] and N2—C15—C14 [110.1 (2)°], which are close to the ideal bond angle of 109.5° for a chair conformation. The sum of the bond angles around N1 (335.73°) indicate *sp* ^3^ hybridization, while the sum of the bond angles around N2 (360°) indicates *sp* ^2^ hybridization. This is also evidenced by the tetra­gonal mol­ecular geometry of C12—N1—C9 [113.89 (18)°], C14—N1—C9 [113.48 (16)°], and C12—N1—C14 [108.36 (16)°] and the trigonal planar mol­ecular geometry of C16—N2—C15 [126.30 (19)°], C16—N2—C13 [120.9 (2)°], and C15—N2—C13 [112.8 (2)°]. The delocalization of the lone pair of N2 into the π bond of carbonyl group causes *sp* ^2^ hybridization of N2.

## Supra­molecular features   

**Figure 4:**
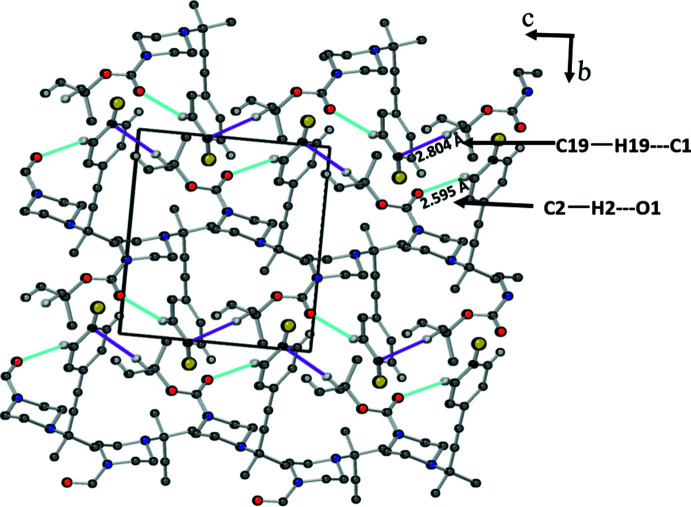
30% probability plot of crystal packing of *tert*-butyl 4-[4-(4-fluoro­phen­yl)-2-methyl­but-3-yn-2-yl]piperazine-1-carboxyl­ate viewed down the *a* axis showing weak hydrogen-bond-like inter­actions between C2—H2⋯O1 and C—H⋯π inter­actions between C19—H19⋯C1 due to dispersion inter­actions. Hydrogen atoms not involved in inter­molecular inter­actions are omitted for clarity.

**Figure 5:**
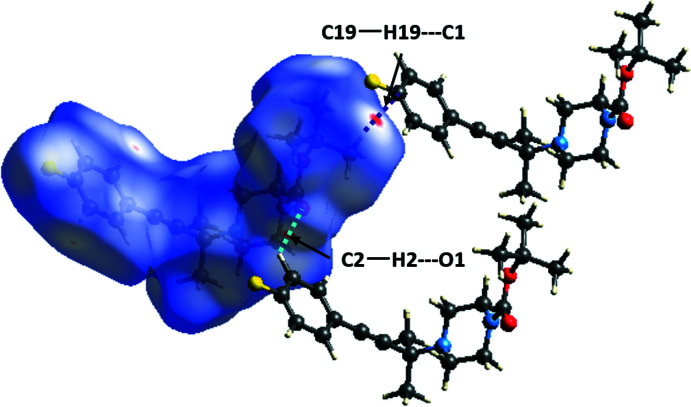
Hirshfeld surface for *tert*-butyl 4-[4-(4-fluoro­phen­yl)-2-methyl­but-3-yn-2-yl]piperazine-1-carboxyl­ate mapped over *d*_norm_ showing weak hydrogen-bond-like inter­actions between C2—H2⋯O1 and C—H⋯π inter­actions between C19—H19⋯C1.

**Figure 6:**
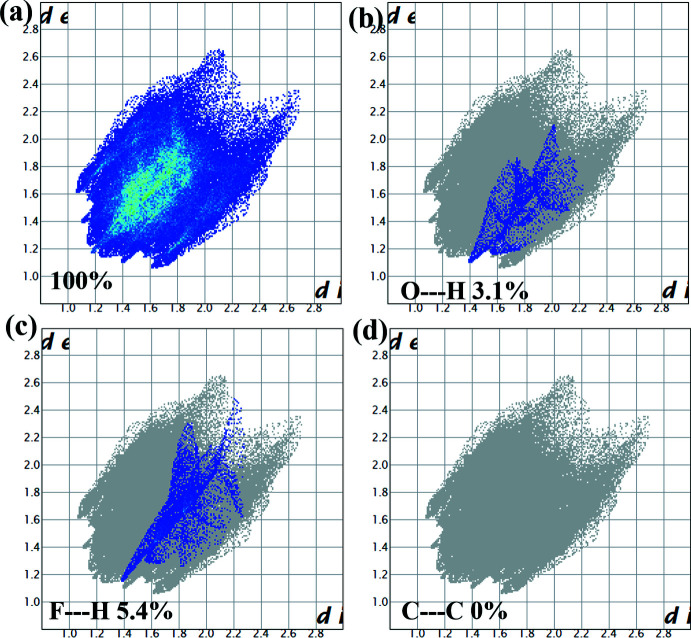
The two-dimensional fingerprint plots of *tert*-butyl 4-[4-(4-fluoro­phen­yl)-2-methyl­but-3-yn-2-yl]piperazine-1-carboxyl­ate showing contributions from different contacts.

Hirshfeld surface analysis and fingerprint analysis were performed using CrystalExplorer (Spackman & Jayatilaka, 2009 ▸, Spackman & McKinnon, 2002 ▸, McKinnon *et al.*, 2007 ▸). In the absence of acidic hydrogen atoms, there cannot be any conventional hydrogen bonds; however, there are directional inter­actions present between C2—H2⋯O1 and C—H⋯π inter­actions between C19—H19⋯C1, as shown in the crystal packing along the *a*-axis (Fig. 4 ▸). These inter­actions are represented by the faint red spots between C2—H2⋯O1 and C19—H19⋯C1 on the Hirshfeld surface mapped over *d*_norm_ in Fig. 5 ▸. The directional C2—H2⋯O1 [*d*(H⋯O) = 2.595 Å] present in the crystal packing could be weak C—H⋯O hydrogen-bond-like inter­actions (Desiraju & Steiner, 1999 ▸) and the C19—H19⋯C1 [*d*(C⋯H) = 2.804 Å] inter­actions could be C—H⋯π inter­actions with dispersion inter­actions as the major source of attraction. Fingerprint analysis (Fig. 6 ▸) complemented the Hirshfeld analysis by showing a minimal contact surface between O⋯H (3.1%) and F⋯H (5.4%), as shown in Fig. 6 ▸ *b* and Fig. 6 ▸ *c*. These could be the directional C—H⋯O inter­actions mentioned previously, and C—H⋯F close contacts attributed to the proximity of the F atom to the C—H⋯π inter­actions. Please see Table 2 ▸ for the inter­atomic contact distances. These data also suggested the absence of π–π stacking as C⋯C contacts contribute 0% of the Hirshfeld surfaces (Fig. 6 ▸ *d*).

## Database survey   

A search in the Cambridge Structural Database (Version 5.41 update of March 2020; (Groom *et al.*, 2016 ▸)) for compounds possessing an *N*-*tert*-butyl piperazine substructure identified 51 compounds. These compounds were several variations of BuckyBall adducts, diketopiperazine derivatives, and ligands. There were only 14 compounds *viz.* DIYWAK (McDermott *et al.*, 2008 ▸), HEHZOL (Legnani *et al.*, 2012 ▸), HICYID, HICYOJ (Sinha *et al.*, 2013*b* ▸), JIFHEO (Zhong *et al.*, 2018 ▸), OFUDAW (Korotaev *et al.*, 2012 ▸), PUYNUS (Jin & Liebscher, 2002 ▸), RIPWUJ (Bobeck *et al.*, 2007 ▸), TILJIJ (Sinha *et al.*, 2013*a* ▸), UPIBIF, UPIBOL (Wiedner & Vedejs, 2010 ▸), UYIHOB (Chen & Cao, 2017 ▸), WANTAJ (Golubev & Krasavin, 2017 ▸), and WINMAH (Brouant & Giorgi, 1995 ▸) that were asymmetrically substituted on the piperazine ring, and none with a synthetically useful second nitro­gen. All were effectively ‘non-inter­mediate’ compounds that could not reasonably serve for additional substitution at the second nitro­gen and none had alkyne substitutions. The quaternary carbon piperazines were explored by Sinha *et al.* (2013*a* ▸,*b* ▸) using an Ugi reaction; however, the present structure is the only compound containing an α,α-dimethyl carbon attached to an alkyne and an amine. This new methodology required the X-ray studies to confirm the generated structure. In summary, to the best of the authors’ knowledge, there is no published crystal structure like the title compound, for a mol­ecule containing asymmetrical substitutions on the piperazine ring, having a synthetically useful second nitro­gen, and an α,α-dimethyl carbon attached to an alkyne and an amine.

## Synthesis and crystallization   

***tert*** **-Butyl 4-(2-cyano­propan-2-yl)piperazine-1-carboxyl­ate (3)**: Ethereal HCl (40.3 mL of a 2.0 *M* in Et_2_O, 80.6 mmol, 1.5 eq. titrated against standardized 1 *N* NaOH to a phenolphthalein pink end-point) was added dropwise to a stirred solution of *tert*-butyl piperazine-1-carboxyl­ate **2** (12.6 g, 53.7 mmol, 1.0 eq.) in MeOH (60 mL) and CH_2_Cl_2_ (60 mL) at 273 K under Argon. The resulting mixture was stirred at 273 K for 1 h, after which the solvent and excess HCl were removed under reduced pressure and the white residual solid was dissolved in water (150 mL). In a well-ventilated fume hood, solid NaCN (2.63 g, 53.7 mmol, 1.0 eq.) and then a solution of acetone (9.4 g, 11.8 mL, 161.2 mmol, 3.0 eq.) in water (20 mL) were added sequentially at room temperature (296 K). The resulting mixture was stirred at room temperature under air for an additional 48 h. Water (100 mL) was added and the mixture was extracted with EtOAc (3 × 100 mL) then NaCl (sat, aq.). The combined organic extracts were dried (MgSO_4_) and the solvent was removed under reduced pressure to give *tert*-butyl 4-(2-cyano­propan-2-yl)piperazine-1-carboxyl­ate **3** as a white crystalline solid, 11 g (64%). MP: 381.2 K (reported 381–383 K) matching the literature (Firth *et al.*, 2016 ▸). ^1^H NMR (400 MHz, CDCl_3_: δ3.50 (*dd*, *J* = 4.8 Hz, 4H), 2.62 (*dd*, *J* = 4.8 Hz, 4H), 1.54 (*s*, 6H), 1.49 (*s*, 9H) matches literature (Firth *et al.*, 2016 ▸).

Note: the aqueous extracts (pH > 10) were collected and the residual cyanide was oxidized to cyanate with sodium hypochlorite (Gerritsen & Margerum, 1990 ▸) and absence of a cyanide ion was confirmed with an MQuant™ Koening Cyanide test indicator from EM sciences.

***tert*** **-Butyl 4-[4-(4-fluoro­phen­yl)-2-methyl­but-3-yn-2-yl]piperazine-1-carboxyl­ate (1)**:

A 250 mL flame-dried, round-bottom flask was cooled under argon and then charged with 1-ethynyl-4-fluoro­benzene **4** (1.98 g, 16.5mmol) in 50 mL of anhydrous THF. This solution was cooled with an external ice-bath. A commercial solution of methyl magnesium bromide (5.25 mL, 16.5 mmol) (Acros, ∼3.2 *M* in THF, assayed against anhydrous diphenyl acetic acid with 2 mg 1,10-phenanthroline as an indicator) was added with slow dropwise addition over 10 minutes. The inter­nal temperature was maintained between 274–275 K. This mixture was stirred at ice-bath temperature for an additional 20 minutes, which resulted in a pale-yellow solution. A solution of *tert*-butyl 4-(2-cyano­propan-2-yl)piperazine-1-carboxyl­ate **3** (Firth *et al.*, 2016 ▸) (2.33 g, 9.2 mmol) in 25 mL THF was added dropwise to this mixture over 10 minutes; the inter­nal temperature was maintained between 274–275.3 K. This deep-yellow solution was permitted to stir with the external ice-bath slowly melting and rising to room temperature, while progress was monitored by TLC (*R*_f_ of product at 0.6 1:1 H:EA, SiO_2_ plates, SWUV and I_2_ visualization). Following stirring for 12 h at 296 K, the crude reaction mixture was cooled to ice-bath temperature and the reaction was quenched with the addition of 10 mL of ice-cold water at a rate of addition that maintained the inter­nal temperature below 278 K. After quenching the organo-base, an additional 50 mL of water were added. Small aliquots of brine and ethanol were used, as required, to break the emulsion in the following extraction. This mixture was extracted with 3 × 20 mL of ethyl acetate, washed (3 × 10 mL H_2_O, 3 × 10 mL brine) dried (Na_2_SO_4_), deca­nted, and the solvent removed under reduced pressure to afford 30.6 g of a yellow solid. This was separated on 50 g of SiO_2_ with hexa­ne/ethyl acetate (1/1) as the eluent to yield *tert*-butyl 4-[4-(4-fluoro­phen­yl)-2-methyl­but-3-yn-2-yl]piperazine-1-carbox­yl­ate **1** as a white powder, 2.74 g (86.3%). This compound was recrystallized from ethyl acetate as colorless plates, having a melting point of 388.1 K. ^1^H NMR (400 MHz, chloro­form-*d*) δ 7.36 (*dd*, *J* = 8.2, 5.6 Hz, 2H), 6.96 (*t*, *J* = 8.5 Hz, 2H), 3.46 (*s*, 5H), 2.63 (*s*, 4H), 1.45 (*s*, 16H). HRMS: (C_20_H_27_FN_2_O_2_) calculated for [*M* + H]^+^ 347.2129, found 347.2127.

## Refinement   

Crystal data, data collection, and structure refinement details are summarized in Table 3 ▸. H atoms were localized in a difference-Fourier map. C-bound H atoms were treated as riding, with C—H = 0.93, 0.96 or 0.97 Å, and with *U*_iso_(H) = 1.2*U*_eq_(C) for aromatic and 1.5*U*_eq_(C) for methyl groups.

## Supplementary Material

Crystal structure: contains datablock(s) I. DOI: 10.1107/S2056989021002346/zl5007sup1.cif

Structure factors: contains datablock(s) I. DOI: 10.1107/S2056989021002346/zl5007Isup3.hkl

Supporting information file. DOI: 10.1107/S2056989021002346/zl5007Isup3.cml

CCDC reference: 2067318

Additional supporting information:  crystallographic information; 3D view; checkCIF report

## Figures and Tables

**Table 1:** Selected geometric parameters (Å, °)

F1—C1	1.359 (3)	N2—C16	1.336 (3)
O1—C16	1.211 (3)	C1—C6	1.351 (4)
O2—C16	1.345 (3)	C7⋯N1	3.508 (3)
			
C12—N1—C14	108.36 (16)	C16—N2—C13	120.9 (2)
C12—N1—C9	113.89 (18)	C15—N2—C13	112.8 (2)
C14—N1—C9	113.48 (16)	N1—C12—C13	110.77 (19)
C16—N2—C15	126.30 (19)	N2—C15—C14	110.1 (2)

**Table 2:** Short inter­atomic contact distances (Å)

Contact	Distance
C2—H2⋯O1	2.595
C19—H19⋯C1	2.804
C19—H19⋯F1	3.163

**Table 3:** Experimental details

Crystal data	
Chemical formula	C_20_H_27_FN_2_O_2_
*M*_r_	346.43
Crystal system, space group	Monoclinic, *P*2_1_
Temperature (K)	293
*a*, *b*, *c* (Å)	10.2576 (11), 9.5127 (10), 10.5318 (11)
β (°)	104.691 (2)
*V* (Å^3^)	994.07 (18)
*Z*	2
Radiation type	Mo *K*α
μ (mm^−1^)	0.08
Crystal size (mm)	0.65 × 0.50 × 0.17

Data collection	
Diffractometer	Bruker SMART APEXII
Absorption correction	Multi-scan (*SADABS*; Sheldrick, 2002 ▸)
*T*_min_, *T*_max_	0.704, 0.746
No. of measured, independent and observed [*I* > 2σ(*I*)] reflections	10640, 5058, 3662
*R*_int_	0.017
(sin θ/λ)_max_ (Å^−1^)	0.675

Refinement	
*R*[*F* ^2^ > 2σ(*F* ^2^)], *wR*(*F* ^2^), *S*	0.040, 0.109, 1.04
No. of reflections	5058
No. of parameters	231
No. of restraints	1
H-atom treatment	H-atom parameters constrained
Δρ_max_, Δρ_min_ (e Å^−3^)	0.12, −0.11

Computer programs: *SMART* and *SAINT* (Bruker, 1998 ▸), *SHELXS97* (Sheldrick, 2008 ▸), *SHELXL2018/3* (Sheldrick, 2015 ▸) and *CrystalMaker* (Palmer, 2014 ▸).

## supplementary crystallographic information

### Crystal data

**Table:**

C_20_H_27_FN_2_O_2_	*F*(000) = 372
*M**_r_* = 346.43	*D*_x_ = 1.157 Mg m^−^^3^
Monoclinic, *P*2_1_	Mo *K*α radiation, λ = 0.71073 Å
*a* = 10.2576 (11) Å	Cell parameters from 3739 reflections
*b* = 9.5127 (10) Å	θ = 2.9–23.9°
*c* = 10.5318 (11) Å	µ = 0.08 mm^−^^1^
β = 104.691 (2)°	*T* = 293 K
*V* = 994.07 (18) Å^3^	Plate, colorless
*Z* = 2	0.65 × 0.50 × 0.17 mm

### Data collection

**Table:**

Bruker SMART APEXII diffractometer	3662 reflections with *I* > 2σ(*I*)
φ and ω Scans scans	*R*_int_ = 0.017
Absorption correction: multi-scan (SADABS; Sheldrick, 2002)	θ_max_ = 28.7°, θ_min_ = 2.0°
*T*_min_ = 0.704, *T*_max_ = 0.746	*h* = −13→13
10640 measured reflections	*k* = −12→12
5058 independent reflections	*l* = −14→14

### Refinement

**Table:**

Refinement on *F*^2^	Primary atom site location: structure-invariant direct methods
Least-squares matrix: full	Secondary atom site location: difference Fourier map
*R*[*F*^2^ > 2σ(*F*^2^)] = 0.040	Hydrogen site location: inferred from neighbouring sites
*wR*(*F*^2^) = 0.109	H-atom parameters constrained
*S* = 1.04	*w* = 1/[σ^2^(*F*_o_^2^) + (0.0543*P*)^2^ + 0.0286*P*] where *P* = (*F*_o_^2^ + 2*F*_c_^2^)/3
5058 reflections	(Δ/σ)_max_ < 0.001
231 parameters	Δρ_max_ = 0.12 e Å^−^^3^
1 restraint	Δρ_min_ = −0.11 e Å^−^^3^

### Special details

**Table:**

Geometry. All esds (except the esd in the dihedral angle between two l.s. planes) are estimated using the full covariance matrix. The cell esds are taken into account individually in the estimation of esds in distances, angles and torsion angles; correlations between esds in cell parameters are only used when they are defined by crystal symmetry. An approximate (isotropic) treatment of cell esds is used for estimating esds involving l.s. planes.

### Fractional atomic coordinates and isotropic or equivalent isotropic displacement parameters (Å^2^)

**Table:**

	*x*	*y*	*z*	*U*_iso_*/*U*_eq_	
F1	1.18988 (19)	0.1239 (2)	−0.11543 (15)	0.1037 (6)	
O1	0.36605 (17)	0.51912 (19)	0.18415 (19)	0.0806 (5)	
O2	0.51029 (17)	0.70053 (17)	0.25327 (19)	0.0744 (5)	
N1	0.75069 (16)	0.3139 (2)	0.49328 (17)	0.0532 (4)	
N2	0.54352 (19)	0.4993 (2)	0.3602 (3)	0.0786 (7)	
C1	1.1321 (3)	0.1417 (3)	−0.0138 (2)	0.0675 (6)	
C2	1.1908 (2)	0.2311 (3)	0.0845 (2)	0.0657 (6)	
H2	1.267513	0.281757	0.081485	0.079*	
C3	1.1337 (2)	0.2446 (3)	0.1888 (2)	0.0607 (5)	
H3	1.173183	0.304622	0.257504	0.073*	
C4	1.0186 (2)	0.1709 (2)	0.1937 (2)	0.0551 (5)	
C5	0.9617 (3)	0.0830 (3)	0.0901 (3)	0.0760 (7)	
H5	0.884011	0.032892	0.090838	0.091*	
C6	1.0188 (3)	0.0687 (4)	−0.0148 (3)	0.0830 (8)	
H6	0.980055	0.010008	−0.084752	0.100*	
C7	0.9619 (2)	0.1869 (3)	0.3042 (2)	0.0623 (5)	
C8	0.9197 (2)	0.1996 (3)	0.3994 (2)	0.0614 (5)	
C9	0.8686 (2)	0.2172 (3)	0.5184 (2)	0.0594 (5)	
C10	0.8289 (3)	0.0738 (3)	0.5623 (3)	0.0761 (7)	
H10A	0.907916	0.016550	0.591129	0.114*	
H10B	0.766913	0.028670	0.490045	0.114*	
H10C	0.786779	0.086116	0.633205	0.114*	
C11	0.9824 (3)	0.2766 (3)	0.6290 (2)	0.0766 (7)	
H11A	1.053887	0.208928	0.652455	0.115*	
H11B	0.948419	0.296461	0.704074	0.115*	
H11C	1.016014	0.361555	0.599756	0.115*	
C12	0.6330 (2)	0.2618 (2)	0.3960 (3)	0.0623 (5)	
H12A	0.612475	0.167143	0.419119	0.075*	
H12B	0.652638	0.258352	0.310685	0.075*	
C13	0.5133 (2)	0.3551 (2)	0.3890 (3)	0.0758 (7)	
H13A	0.437271	0.321130	0.321152	0.091*	
H13B	0.488758	0.352041	0.472069	0.091*	
C14	0.7810 (2)	0.4556 (2)	0.4548 (2)	0.0629 (6)	
H14A	0.799613	0.452268	0.369024	0.075*	
H14B	0.860821	0.490898	0.517127	0.075*	
C15	0.6649 (2)	0.5530 (3)	0.4505 (3)	0.0753 (7)	
H15A	0.650242	0.561673	0.537584	0.090*	
H15B	0.685611	0.645571	0.422237	0.090*	
C16	0.4649 (2)	0.5686 (2)	0.2590 (2)	0.0613 (5)	
C17	0.4383 (3)	0.8005 (3)	0.1535 (2)	0.0719 (6)	
C18	0.4377 (4)	0.7509 (5)	0.0174 (3)	0.1233 (14)	
H18A	0.411731	0.827031	−0.043585	0.185*	
H18B	0.374709	0.674936	−0.007262	0.185*	
H18C	0.526238	0.719043	0.016399	0.185*	
C19	0.2976 (3)	0.8232 (4)	0.1690 (3)	0.0933 (9)	
H19A	0.255882	0.899276	0.113429	0.140*	
H19B	0.301797	0.845773	0.258749	0.140*	
H19C	0.245633	0.739092	0.144516	0.140*	
C20	0.5234 (5)	0.9306 (4)	0.1912 (4)	0.1246 (14)	
H20A	0.487111	1.005437	0.131485	0.187*	
H20B	0.614143	0.911229	0.187209	0.187*	
H20C	0.523167	0.958052	0.278851	0.187*	

### Atomic displacement parameters (Å^2^)

**Table:**

	*U*^11^	*U*^22^	*U*^33^	*U*^12^	*U*^13^	*U*^23^
F1	0.1100 (12)	0.1444 (17)	0.0629 (8)	0.0197 (12)	0.0335 (8)	−0.0031 (9)
O1	0.0619 (9)	0.0604 (10)	0.1066 (13)	−0.0065 (8)	−0.0025 (9)	0.0007 (9)
O2	0.0717 (10)	0.0445 (8)	0.0986 (12)	−0.0040 (8)	0.0064 (9)	0.0062 (8)
N1	0.0502 (9)	0.0478 (9)	0.0611 (9)	0.0025 (7)	0.0132 (7)	0.0059 (8)
N2	0.0513 (10)	0.0470 (11)	0.1223 (17)	−0.0065 (8)	−0.0061 (11)	0.0149 (11)
C1	0.0731 (15)	0.0785 (16)	0.0515 (12)	0.0157 (13)	0.0170 (11)	0.0065 (11)
C2	0.0561 (12)	0.0724 (16)	0.0695 (14)	0.0003 (11)	0.0173 (11)	−0.0018 (12)
C3	0.0586 (12)	0.0612 (13)	0.0615 (13)	0.0023 (10)	0.0136 (10)	−0.0077 (10)
C4	0.0534 (11)	0.0551 (12)	0.0558 (11)	0.0101 (9)	0.0119 (9)	0.0081 (9)
C5	0.0692 (14)	0.0772 (17)	0.0781 (16)	−0.0132 (13)	0.0125 (12)	−0.0043 (13)
C6	0.0916 (19)	0.0891 (19)	0.0612 (14)	−0.0063 (16)	0.0063 (13)	−0.0167 (13)
C7	0.0591 (12)	0.0619 (13)	0.0654 (13)	0.0095 (10)	0.0151 (10)	0.0103 (10)
C8	0.0592 (12)	0.0600 (13)	0.0664 (13)	0.0088 (10)	0.0181 (10)	0.0108 (11)
C9	0.0569 (11)	0.0610 (13)	0.0618 (12)	0.0105 (10)	0.0176 (9)	0.0125 (10)
C10	0.0811 (16)	0.0624 (15)	0.0913 (18)	0.0187 (13)	0.0337 (14)	0.0265 (13)
C11	0.0660 (14)	0.095 (2)	0.0641 (14)	0.0166 (13)	0.0075 (11)	0.0112 (13)
C12	0.0601 (12)	0.0428 (10)	0.0788 (14)	−0.0063 (9)	0.0080 (10)	0.0050 (10)
C13	0.0522 (12)	0.0513 (14)	0.115 (2)	−0.0074 (10)	0.0041 (13)	0.0170 (13)
C14	0.0513 (11)	0.0507 (12)	0.0793 (14)	−0.0079 (9)	0.0028 (10)	0.0037 (11)
C15	0.0606 (13)	0.0452 (12)	0.1076 (19)	−0.0031 (10)	−0.0019 (13)	−0.0023 (12)
C16	0.0480 (11)	0.0445 (11)	0.0910 (16)	0.0004 (9)	0.0165 (11)	−0.0030 (11)
C17	0.0970 (17)	0.0551 (13)	0.0653 (13)	0.0022 (13)	0.0237 (12)	0.0101 (11)
C18	0.165 (4)	0.137 (3)	0.084 (2)	−0.021 (3)	0.063 (2)	−0.012 (2)
C19	0.103 (2)	0.0772 (18)	0.098 (2)	0.0310 (18)	0.0233 (17)	0.0133 (16)
C20	0.168 (4)	0.0650 (19)	0.130 (3)	−0.031 (2)	0.018 (3)	0.0250 (19)

### Geometric parameters (Å, º)

**Table:**

F1—C1	1.359 (3)	C10—H10C	0.9600
O1—C16	1.211 (3)	C11—H11A	0.9600
O2—C16	1.345 (3)	C11—H11B	0.9600
O2—C17	1.470 (3)	C11—H11C	0.9600
N1—C12	1.457 (3)	C12—C13	1.502 (3)
N1—C14	1.463 (3)	C12—H12A	0.9700
N1—C9	1.489 (3)	C12—H12B	0.9700
N2—C16	1.336 (3)	C13—H13A	0.9700
N2—C15	1.454 (3)	C13—H13B	0.9700
N2—C13	1.456 (3)	C14—C15	1.501 (4)
C1—C6	1.351 (4)	C14—H14A	0.9700
C1—C2	1.357 (4)	C14—H14B	0.9700
C2—C3	1.376 (3)	C15—H15A	0.9700
C2—H2	0.9300	C15—H15B	0.9700
C3—C4	1.385 (3)	C17—C18	1.508 (4)
C3—H3	0.9300	C17—C19	1.508 (4)
C4—C5	1.381 (3)	C17—C20	1.509 (4)
C4—C7	1.434 (3)	C18—H18A	0.9600
C5—C6	1.382 (4)	C18—H18B	0.9600
C5—H5	0.9300	C18—H18C	0.9600
C6—H6	0.9300	C19—H19A	0.9600
C7—C8	1.195 (3)	C19—H19B	0.9600
C8—C9	1.486 (3)	C19—H19C	0.9600
C9—C10	1.528 (4)	C20—H20A	0.9600
C9—C11	1.532 (3)	C20—H20B	0.9600
C10—H10A	0.9600	C20—H20C	0.9600
C10—H10B	0.9600		
			
C16—O2—C17	121.26 (19)	N1—C12—H12B	109.5
C12—N1—C14	108.36 (16)	C13—C12—H12B	109.5
C12—N1—C9	113.89 (18)	H12A—C12—H12B	108.1
C14—N1—C9	113.48 (16)	N2—C13—C12	110.6 (2)
C16—N2—C15	126.30 (19)	N2—C13—H13A	109.5
C16—N2—C13	120.9 (2)	C12—C13—H13A	109.5
C15—N2—C13	112.8 (2)	N2—C13—H13B	109.5
C6—C1—C2	122.8 (2)	C12—C13—H13B	109.5
C6—C1—F1	118.4 (2)	H13A—C13—H13B	108.1
C2—C1—F1	118.8 (2)	N1—C14—C15	110.74 (18)
C1—C2—C3	118.1 (2)	N1—C14—H14A	109.5
C1—C2—H2	120.9	C15—C14—H14A	109.5
C3—C2—H2	120.9	N1—C14—H14B	109.5
C2—C3—C4	121.5 (2)	C15—C14—H14B	109.5
C2—C3—H3	119.3	H14A—C14—H14B	108.1
C4—C3—H3	119.3	N2—C15—C14	110.1 (2)
C5—C4—C3	118.0 (2)	N2—C15—H15A	109.6
C5—C4—C7	121.9 (2)	C14—C15—H15A	109.6
C3—C4—C7	120.1 (2)	N2—C15—H15B	109.6
C4—C5—C6	120.7 (2)	C14—C15—H15B	109.6
C4—C5—H5	119.6	H15A—C15—H15B	108.1
C6—C5—H5	119.6	O1—C16—N2	124.3 (2)
C1—C6—C5	118.8 (2)	O1—C16—O2	125.2 (2)
C1—C6—H6	120.6	N2—C16—O2	110.50 (19)
C5—C6—H6	120.6	O2—C17—C18	110.9 (3)
C8—C7—C4	177.4 (2)	O2—C17—C19	109.7 (2)
C7—C8—C9	179.2 (3)	C18—C17—C19	112.0 (3)
C8—C9—N1	111.34 (17)	O2—C17—C20	101.0 (2)
C8—C9—C10	109.5 (2)	C18—C17—C20	111.7 (3)
N1—C9—C10	109.80 (17)	C19—C17—C20	111.1 (3)
C8—C9—C11	108.59 (18)	C17—C18—H18A	109.5
N1—C9—C11	109.55 (19)	C17—C18—H18B	109.5
C10—C9—C11	108.0 (2)	H18A—C18—H18B	109.5
C9—C10—H10A	109.5	C17—C18—H18C	109.5
C9—C10—H10B	109.5	H18A—C18—H18C	109.5
H10A—C10—H10B	109.5	H18B—C18—H18C	109.5
C9—C10—H10C	109.5	C17—C19—H19A	109.5
H10A—C10—H10C	109.5	C17—C19—H19B	109.5
H10B—C10—H10C	109.5	H19A—C19—H19B	109.5
C9—C11—H11A	109.5	C17—C19—H19C	109.5
C9—C11—H11B	109.5	H19A—C19—H19C	109.5
H11A—C11—H11B	109.5	H19B—C19—H19C	109.5
C9—C11—H11C	109.5	C17—C20—H20A	109.5
H11A—C11—H11C	109.5	C17—C20—H20B	109.5
H11B—C11—H11C	109.5	H20A—C20—H20B	109.5
N1—C12—C13	110.77 (19)	C17—C20—H20C	109.5
N1—C12—H12A	109.5	H20A—C20—H20C	109.5
C13—C12—H12A	109.5	H20B—C20—H20C	109.5
			
C6—C1—C2—C3	1.7 (4)	C16—N2—C13—C12	125.9 (3)
F1—C1—C2—C3	−177.9 (2)	C15—N2—C13—C12	−53.4 (3)
C1—C2—C3—C4	−0.7 (3)	N1—C12—C13—N2	56.6 (3)
C2—C3—C4—C5	−0.3 (3)	C12—N1—C14—C15	60.9 (2)
C2—C3—C4—C7	179.7 (2)	C9—N1—C14—C15	−171.60 (19)
C3—C4—C5—C6	0.5 (4)	C16—N2—C15—C14	−125.5 (3)
C7—C4—C5—C6	−179.6 (2)	C13—N2—C15—C14	53.7 (3)
C2—C1—C6—C5	−1.5 (4)	N1—C14—C15—N2	−57.5 (3)
F1—C1—C6—C5	178.1 (2)	C15—N2—C16—O1	178.3 (3)
C4—C5—C6—C1	0.4 (4)	C13—N2—C16—O1	−0.9 (4)
C12—N1—C9—C8	64.1 (2)	C15—N2—C16—O2	−2.3 (4)
C14—N1—C9—C8	−60.5 (2)	C13—N2—C16—O2	178.6 (2)
C12—N1—C9—C10	−57.3 (2)	C17—O2—C16—O1	1.8 (4)
C14—N1—C9—C10	178.12 (19)	C17—O2—C16—N2	−177.7 (2)
C12—N1—C9—C11	−175.76 (19)	C16—O2—C17—C18	−63.8 (3)
C14—N1—C9—C11	59.6 (2)	C16—O2—C17—C19	60.4 (3)
C14—N1—C12—C13	−60.2 (2)	C16—O2—C17—C20	177.7 (3)
C9—N1—C12—C13	172.46 (18)		

### Selected bond lengths (Å) and bond angles (°)

**Table:**

F1—C1	1.359 (3)
O1—C16	1.211 (3)
O2—C16	1.345 (3)
N2—C16	1.336 (3)
C1—C6	1.351 (4)
C7—N2	3.508
N1—C12—C13	110.77 (19)
N2—C15—C14	110.1 (2)
C12—N1—C9	113.89 (18)
C14—N1—C9	113.48 (16)
C12—N1—C14	108.36 (16)
C16—N2—C15	126.30 (19)
C16—N2—C13	120.9 (2)
C15—N2—C13	112.8 (2)
